# In vitro screening antiviral activity of Thai medicinal plants against porcine reproductive and respiratory syndrome virus

**DOI:** 10.1186/s12917-020-02320-8

**Published:** 2020-03-30

**Authors:** Chaiwat Arjin, Kidsadagon Pringproa, Surat Hongsibsong, Warintorn Ruksiriwanich, Mintra Seel-audom, Supamit Mekchay, Korawan Sringarm

**Affiliations:** 1 Department of Animal and Aquatic Sciences, Faculty of Agriculture, Chiang Mai University, 239, Huaykaew Road, Suthep, Muang, Chiang Mai, 50200 Thailand; 2 Department of Veterinary Bioscience and Veterinary Public Health, Faculty of Veterinary Medicine, Chiang Mai University, Chiang Mai, 50100 Thailand; 3 Cluster of Research and Development of Pharmaceutical and Natural Products Innovation for Human or Animal, Chiang Mai University, Chiang Mai, 50200 Thailand; 4 Environment and Health Research Unit, Research Institute for Health Sciences, Chiang Mai University, Chiang Mai, 50200 Thailand; 5 Department of Pharmaceutical Sciences, Faculty of Pharmacy, Chiang Mai University, Chiang Mai, 50200 Thailand

**Keywords:** Porcine reproductive and respiratory syndrome, Porcine reproductive and respiratory syndrome virus, Antiviral activity, Thai medicinal plants

## Abstract

**Background:**

Porcine reproductive and respiratory syndrome (PRRS) caused by PRRS virus (PRRSV) results in economic losses in the swine industry globally. Several studies have investigated the use of plant extracts in the prevention and control of PRRS outbreaks. Thai medicinal plants may be useful for treating PRRSV infection in pigs. Therefore, we investigated the in vitro anti-PRRSV and antioxidant properties of seven Thai medicinal plants: *Caesalpinia sappan* Linn., *Garcinia mangostana* Linn*.*, *Houttuynia cordata*, *Perilla frutescens*, *Clinacanthus nutans*, *Phyllanthus emblica*, and *Tiliacora triandra*.

**Results:**

Using antiviral screening, we observed that *T. triandra* extract strongly inhibited PRRSV infectivity in MARC-145 cells [virus titer 3.5 median tissue culture infective dose (TCID_50_)/ml (log10)] at 24 h post-infection, whereas *C. sappan* extract strongly inhibited PRRSV replication [virus titer 2.5 TCID_50_/ml (log10)] at 72 h post-infection. *C. sappan* extract had the highest total phenolic content [220.52 mM gallic acid equivalent/g] and lowest half-maximal inhibitory concentration [1.17 mg/ml in 2,2-diphenyl-1-picrylhydrazyl and 2.58 mg/ml in 2,2-azino-bis (3-ethylbenzothiazo-line-6-sulfonic acid) diammonium salt].

**Conclusion:**

*T. triandra* extract could inhibit PRRSV infectivity, whereas *C. sappan* extract was the most effective in inhibiting PRRSV replication in MARC-145 cells. This study elucidates the antiviral activities of Thai medicinal plant extracts in vivo. The results promise that Thai medicinal plant extracts, particularly *T. triandra* and *C. sappan* extracts, can be developed into pharmaceutical drugs for the prevention of PRRS in pigs.

## Background

Porcine reproductive and respiratory syndrome virus (PRRSV) is endemic in most pig-producing countries, and it results in enormous economic losses to the swine industry globally [1]. This enveloped, positive-sense, single-stranded RNA virus belongs to the *Arteriviridae* family (order *Nidovirales*), which also includes the equine arteritis virus, mouse lactate dehydrogenase-elevating virus, and simian hemorrhagic fever virus [2]. In general, PRRSV infection causes a disease that is characterized by reproductive failure in sows and respiratory infections in growing pigs [3], and this disease predisposes pigs to infection by bacteria and other viral pathogens [4, 5]. This disease is known as porcine reproductive and respiratory syndrome (PRRS) and has become endemic in many countries throughout the world following an epidemic phase [6, 7]. Its incidence was first reported in Thailand in 1989, and since then, several outbreaks have been reported [8]. It has become a major infectious disease that causes high mortality in swine and production losses in the swine industry in this country.

Preventative measures such as gilt acclimatization, vigilant biosecurity, and vaccination have been shown to be useful in controlling PRRS outbreaks, and supportive treatments are available for alleviating its severity; however, no specific treatment for PRRS is available [9, 10]. Antiviral therapeutics are a critical tool for combating viral infections, particularly in cases wherein no vaccines are available against the circulating virus. Thus, pharmacological intervention may represent an alternative approach in controlling PRRSV. A number of natural compounds and compositions have been shown to possess antiviral activities against PRRSV. Gao et al. [11] showed that *Cryptoporus volvatus* extract exhibited antiviral activity against PRRSV infection and replication. Pringproa et al. [12] reported that crude *Cynodon dactylon* extract significantly inhibited PRRSV replication as early as 24 h post-infection (hpi). Therefore, the antiviral activities of other Thai medicinal plants against PRRSV should also be investigated. Thai medicinal plants such as *Caesalpinia sappan* Linn., *Garcinia mangostana* Linn., *Houttuynia cordata*, *Perilla frutescens*, *Clinacanthus nutans*, *Phyllanthus emblica*, and *Tiliacora triandra* are known to have antioxidant and antiviral activities. These plants have already been promoted for use in primary health care and have been classified according to their pharmacological actions [13–18]. Therefore, the aim of this study was to determine the antiviral activities of Thai medicinal plant extracts against PRRSV infection in vitro and to measure their phytochemical contents to develop an alternative anti-PRRSV therapy for use in veterinary medicine.

## Results

### Cytotoxic activities of plant extracts

**Fig. 1:**
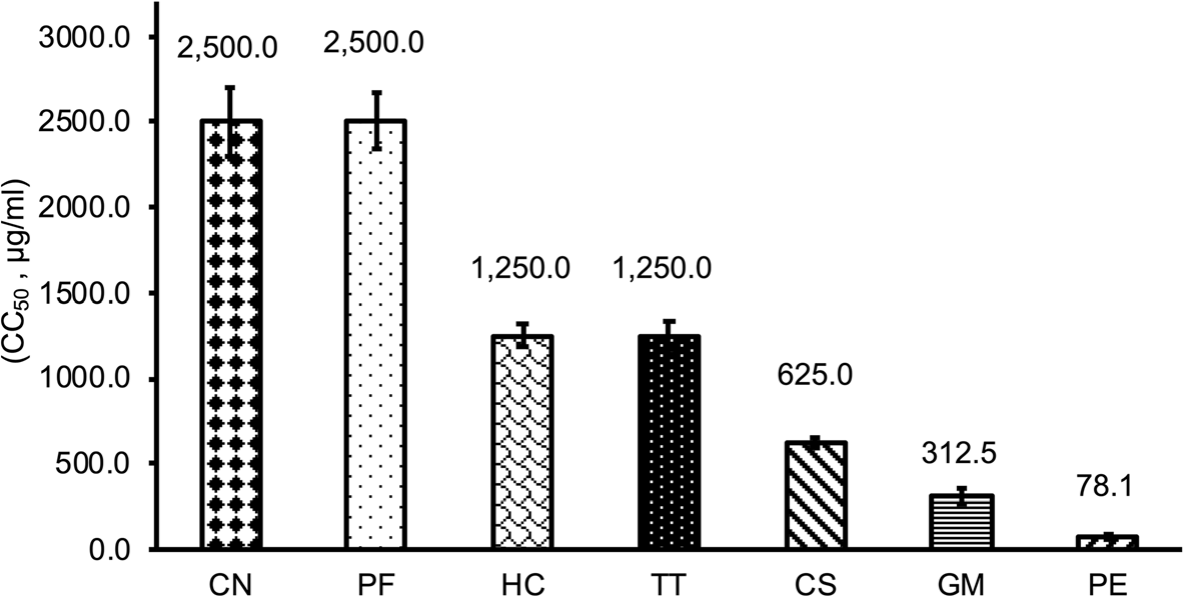
Cytotoxity of the seven Thai medicinal plant extracts on MARC-145 cells determined by the 3-(4,5-dimethylthiazol-2-yl)-2,5-diphenyl tetrazolium bromide (MTT) assay. MARC-145 cells were incubated with various concentrations of these plant extracts or control without plant extract for 72 h prior to the MTT assay. Values are expressed as mean ± standard error. CN, *Clinacanthus nutans*; PF, *Perilla frutescens*; HC, *Houttuynia cordata*; TT, *Tiliacora triandra*; CS, *Caesalpinia sappan* Linn.; GM, *Garcinia mangostana* Linn.; PE, *Phyllanthus emblica*; CC_50_, 50% cytotoxic concentration

Prior to determining antiviral activity, we evaluated the cytotoxicity of the seven Thai medicinal plant extracts on the viability of MARC-145 cells, and viability is expressed as 50% cytotoxic concentration (CC_50_). The results showed that the CC_50_ of the seven plant extracts ranged from 78 to 2500 μg/ml, and the effect of Thai medicinal plant extract concentration on the tested cells increased in a dose-dependent manner (Fig. 1). *P. emblica* extract had the lowest CC_50_ of 78 μg/ml. The CC_50_ of *G. mangostana* extract was the second lowest (312.5 μg/ml) and that of *C. sappan* extract was 625 μg/ml. Further, *T. triandra* and *H. cordata* extracts had CC_50_ of 1250 μg/ml, whereas *C. nutans* and *P. frutescens* extracts had the highest CC_50_ (2500 μg/ml).

### Inhibition of PRRSSV infection by Thai medicinal plant extracts

**Fig. 2:**
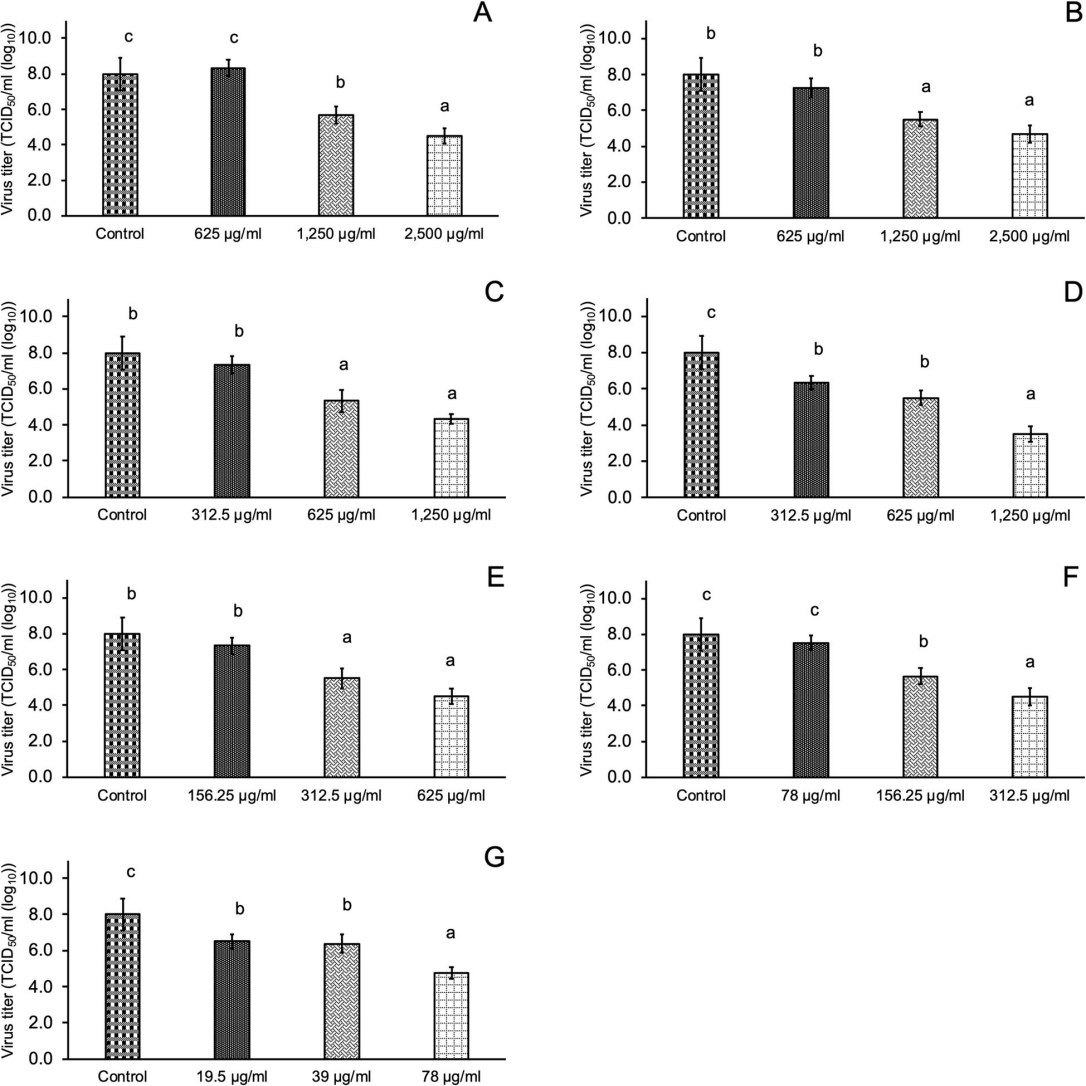
Virus titer for the inhibition of PRRSV infectivity of seven Thai medicinal plant extracts at 24 h post-infection (hpi). **A***Clinacanthus nutans*; **B***Perilla frutescens*; **C***Houttuynia cordata*; **D***Tiliacora triandra*; **E***Caesalpinia sappan* Linn.; **F***Garcinia mangostana* Linn. and **G***Phyllanthus emblica*. a, b, and c, *P*-value of < 0.05 compared with different concentrations of the plant extracts

**Fig. 3:**
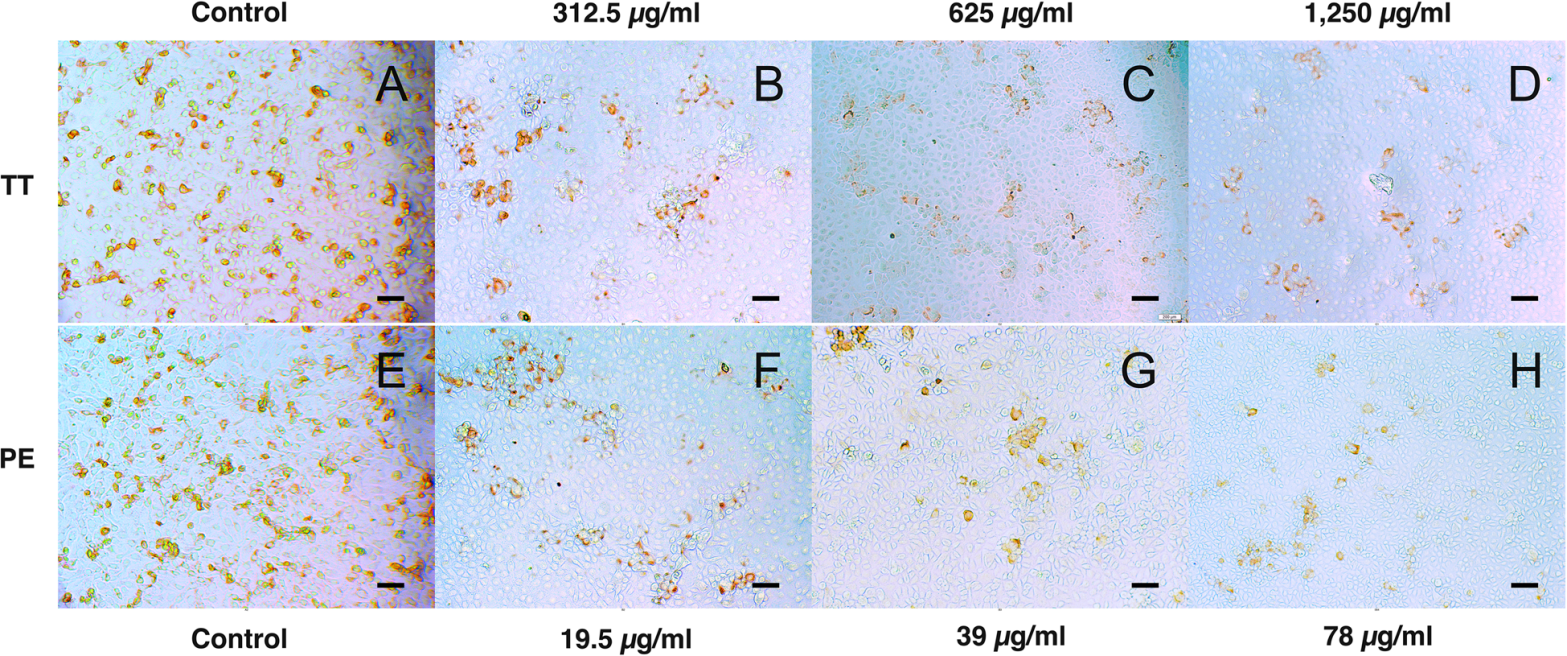
Immunoperoxidase monolayer assay (IPMA) showing the inhibition of PRRSV infection in MARC-145 cells by *Tiliacora triandra* (TT) extract at concentrations of 312.5, 625, and 1250 μg/ml (**a–d**) and *P. emblica* (PE) extract at concentrations of 19.5, 39, and 78 μg/ml (**e–h**). Scale bar in the figure: 200 *μ*m

We treated PRRSV with different concentrations of Thai medicinal plant extracts that were determined based on their CC_50_ values so that these plant extracts did not affect the proliferative activity of MARC-145 cells. The screening results of the inhibition of PRRSV infectivity showed the potential of Thai medicinal plant extracts to inhibit PRRSV infectivity (Fig. 2). *T. triandra* extract significantly inhibited PRRSV infectivity in MARC-145 cells at 24 hpi when supplied at a concentration of 1250 μg/ml (*P* < 0.05), and the observed virus titer at this concentration was 3.5 TCID_50_/ml (log_10_). Interestingly, *P. emblica* extract at a low concentration of 78 μg/ml could inhibit PRRSV infectivity [virus titer = 4.5 TCID_50_/ml (log_10_)]. As shown in Fig. 3, immunoperoxidase monolayer assay (IPMA) indicated that *T. triandra* and *P. emblica* extracts blocked PRRSV infectivity in MARC-145 cells, as shown by slight brown staining of cells.

### Thai medicinal plant extracts inhibit PRRSV replication

**Fig. 4:**
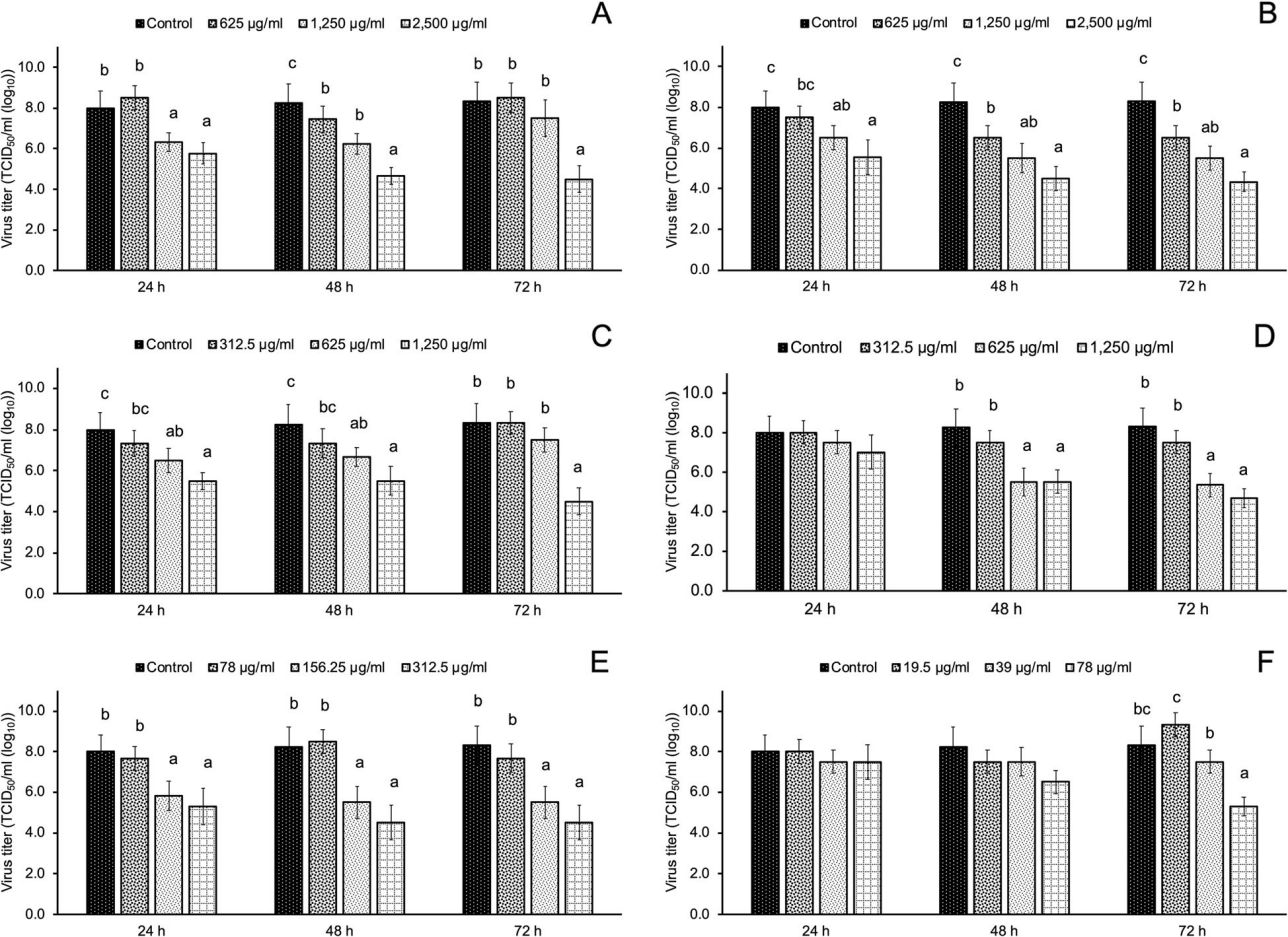
Virus titer for the inhibition of PRRSV replication of seven Thai medicinal plant extracts at 24, 48, and 72 h post-infection (hpi). **A***Clinacanthus nutans*; **B***Perilla frutescens*; **C***Houttuynia cordata*; **D***Tiliacora triandra*; **E***Garcinia mangostana* Linn. and **F***Phyllanthus emblica*. a, b, and c; *P*-value of < 0.05 compared with different concentrations of the plant extracts

**Fig. 5:**
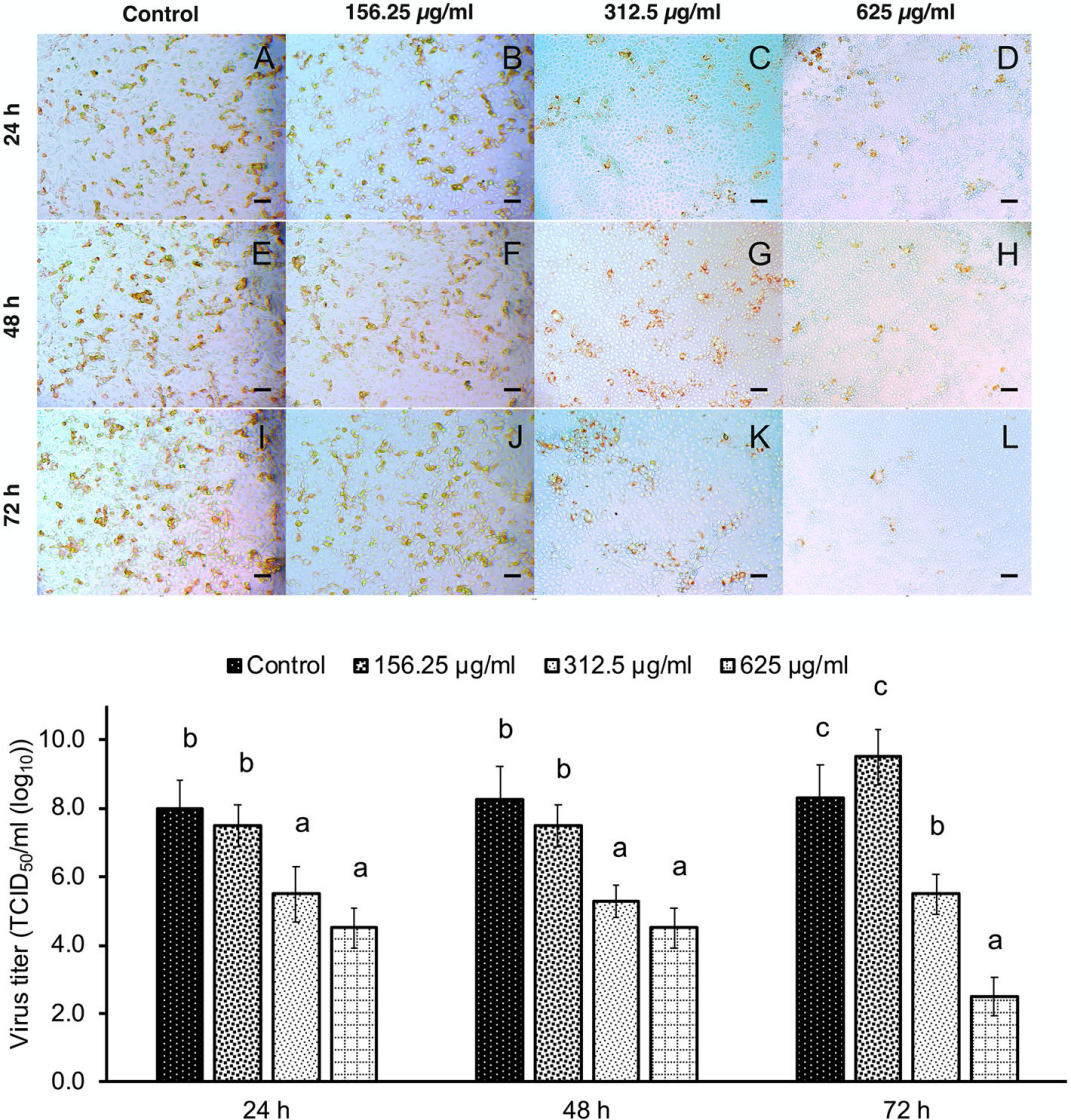
IPMA of *Caesalpinia sappan* Linn. inhibiting PRRSV replication in MARC-145 cells at 24 (**A–D**), 48 (**E** and **F**), and 72 h post-infection (hpi) (**I–L**). a, b, and c: *P*-value of < 0.05 compared with different concentrations of *C. sappan*. Scale bar in the figure: 200 *μ*m

Different Thai medicinal plant extracts were tested in an in vitro inhibitor screening assay to determine inhibition of PRRSV replication at three time intervals (24, 48, and 72 hpi). At various time points after the infection, PRRSV in supernatants was quantified for determining virus titer by IPMA. Results of screening were the same as those of the inhibition test of PRRSV infectivity, i.e., PRRSV replication was inhibited in a dose-dependent manner (Fig. 4). Interestingly, as shown in Fig. 5, we found that *C. sappan* extract had significant potential to inhibit PRRSV replication in vitro. As shown in Fig. 5L, few cells that were stained brown showed the efficiency of *C. sappan* extract at a concentration of 625 μg/ml, and the inhibition of PRRSV replication by *C. sappan* extract was significantly stronger than that by other plant extracts at 72 hpi [2.7 TCID_50_/ml (log_10_)].

### Phytochemical contents of Thai medicinal plant extracts

The total phenolic contents of the seven Thai medicinal plant extracts were determined using the Folin–Ciocalteu assay by constructing a standard curve of gallic acid. Total phenolic content was the highest in *C. sappan* extract [mean ± standard error: 220.52 ± 4.47 mM gallic acid equivalent (GAE)/g sample], followed by *G. mangostana* extract (91.16 ± 4.62 mM GAE/g sample), with the lowest total phenolic content was observed in *H. cordata* extract (8.51 ± 0.04 mM GAE/g sample) (Table 1).

**Table 1:** Total phenolic contents and antioxidant activities of seven Thai medicinal plant extracts

	Total phenolic (mM GAE/g)	DPPH (IC_50_, mg/ml)	ABTS (IC_50_, mg/ml)	FRAP (mM Fe^2+^/g)
*Caesalpinia sappan*	220.52 ± 4.47	1.17 ± 0.06	2.57 ± 0.16	334.78 ± 13.15
*Garcinia mangostana*	91.16 ± 4.62	4.82 ± 0.58	4.98 ± 0.10	46.12 ± 1.27
*Houttuynia cordata*	14.25 ± 0.20	97.79 ± 4.14	72.02 ± 4.01	8.55 ± 0.18
*Perilla frutescens*	29.86 ± 0.41	11.68 ± 0.51	21.37 ± 1.28	43.32 ± 0.92
*Clinacanthus nutans*	25.52 ± 0.22	50.34 ± 5.60	37.82 ± 1.25	18.39 ± 0.54
*Phyllanthus emblica*	44.35 ± 0.24	3.49 ± 0.17	4.95 ± 0.11	94.17 ± 0.62
*Tiliacora triandra*	30.45 ± 1.51	17.77 ± 0.22	21.16 ± 1.06	30.58 ± 1.13

*DPPH* 2,2-diphenyl-1-picrylhydrazyl, *ABTS* 2,2-azino-bis (3-ethylbenzothiazo-line-6-sulfonic acid) diammonium salt, *FRAP* ferric reducing antioxidant power, *GAE* gallic acid equivalents, *IC*_*50*_ half maximal inhibitory concentration

### Antioxidant activity

*C. sappan* extract had the highest antioxidant activity, with IC_50_ values of 1.17 ± 0.06 mg/ml in 2,2-diphenyl-1-picrylhydrazyl (DPPH) and 2.57 ± 0.16 mg/ml n 2,2-azino-bis(3-ethylbenzothiazo-line-6-sulfonic acid) diammonium salt (ABTS) and a reducing power of 334.78 ± 13.15 mM Fe^2+^/g in the ferric-reducing antioxidant power (FRAP) assay (Table 1). *P. emblica* extract had the second strongest antioxidant activity against free radicals, with IC_50_ values of 3.49 ± 0.17 mg/ml in DPPH and 4.95 ± 0.11 mg/ml in ABTS and a reducing power of 94.17 ± 0.62 mM Fe^2+^/g sample in the FRAP assay.

## Discussion

PRRSV outbreak causes significant economic loss in the swine industry worldwide. The current commercial PRRSV vaccines are inadequate to protect pigs from PRRSV infections [19]. Medicinal plants have progressively been explored as suitable alternative sources of antiviral agents [20]. Thai medicinal plants have widely been used as a source of herbal medicines because of their high bioactive compound contents that are effective against various diseases. In this study, seven Thai medicinal plant extracts were screened for their antiviral activity against PRRSV.

Before determining the antiviral properties of a compound, it is essential that a cytotoxicity assay is performed to determine the concentrations that can be used to avoid cell damage and ensure PRRSV selectivity in vitro. In this study, we reported cytotoxicity as CC_50_, which indicates the concentration of a substance that can inhibit virus activity by 50%. We found that *P. emblica* extract showed the highest cell toxicity (78.1 μg/ml). In this study, high-potential plant extracts were found to be *C. sappan* and *T. triandra* extracts, with CC_50_ of 625 and 1250 μg/ml, respectively. Antiviral compounds should be highly effective while showing minimal toxicity to normal cells and tissues [21].

In this study, we investigated the antiviral activity of seven Thai medicinal plant extracts against PRRSV by assessing the inhibition of PRRSV infection and replication in MARC-145 cells. The range of plant extract concentrations was determined based on their CC_50_ values. *P. emblica* extract inhibited PRRSV infection in MARC-145 cells and in vitro. *P. emblica* extract at a concentration of 78 μg/ml inhibited PRRSV infectivity at a virus titer of 4.5 TCID_50_/ml (log_10_). In this study, *P. emblica* extract showed the highest cytotoxicity to MARC-145 cells with CC_50_ of < 100 μg/ml. Therefore, the antiviral activity of other plant extracts were investigated in this study. We found that *T. triandra* extract at a concentration of 1250 μg/ml significantly inhibited PRRSV infectivity at a virus titer of 3.5 TCID_50_ (log10). While *T. triandra* extract has been used as anti-inflammatory [22], anticancer [23], and antimicrobial agents against *Mycobacterium tuberculosis* [24], its antiviral activity, particularly against PRRSV, has not been investigated previously. Therefore, this is the first report to indicate that *T. triandra* extract could significantly prevent the entry of PRRSV into MARC-145 cells. However, *T. triandra* extract was not found to be effective in inhibiting PRRSV replication. All studied plant extracts could inhibit PRRSV replication when applied at high concentrations, as shown by the linear regression model from 24 to 72 hpi after incubation with PRRSV. *C*. *sappan* extract at a concentration of 625 μg/ml could inhibit PRRSV replication as 72 hpi [virus titer 2.7 TCID_50_ (log10)]. Although the antiviral activity of *C. sappan* extract against the influenza virus [13] and the antimicrobial properties of *C. sappan* [25] have previously been investigated, this is the first study to reveal the inhibitory activity of *C. sappan* extract on PRRSV replication in MARC-145 cells.

Regarding phytochemical content, *C. sappan* extract had the highest total phenolic content (220.52 ± 4.47 mM GAE/g sample). The total phenolic content of a plant is considered an indicator of its antioxidant capacity because the redox properties of phenolic compounds allow them to act as reducing agents, hydrogen donors, and radical scavengers [22]. Previously, Lee et al. [26] reported that ethanolic *C. sappan* extract had a total phenolic content of 723.67 μg GAE/mg. The values of total phenolic content in this study were slightly different from those reported previously. This may be because of the different durations, geographical variations, or extraction methods, which may have altered the phenolic content. Ethanolic plant extracts can be used for the investigation of antiviral activity in a cell line. Abu-Jafar and Huleihel [27] reported that ethanolic *Eucalyptus camaldulensis* leave extracts had strong antiviral activity against different members of the herpes virus family (HSV-1, HSV-2, and VZV). Ramalingam et al. [28] reported that the ethanolic extracts of *Andrographis paniculata* have the highest antiviral inhibitory effects against dengue virus in Vero cells.

The screening of plants as possible sources of antiviral agents has led to the discovery of potent inhibitors of in vitro viral replication, thereby increasing the probability of identifying new bioactive plant compounds [29]. These findings suggest the appropriate species and concentration of plant extract that could effectively inhibit PRRSV replication, with both *T. triandra* and *C. sappan* extracts being highly effective in inhibiting PRRSV infection in vitro by interfering with viral attachment and inhibiting viral replication and/or virus release, respectively. The modes of action of *T. triandra* and *C. sappan* extracts against PPRSV require further investigation but are likely to be related to the natural compounds they contain. Therefore, it was speculated that both *T. triandra* and *C. sappan* extracts are potential candidates for preventing PRRSV infection in pigs. However, the plant extracts used for testing antiviral activity was crude extracts. In future, we plan to purify the most effective Thai medicinal plant extracts (*T. triandra* and *C. sappan* extracts) for screening the active compound that is highly effective against PRRSV.

## Conclusion

Thai medicinal plant extracts exhibit antiviral activity against PRRSV. *T*. *triandra* extract effectively inhibited PRRSV infection. and *C. sappan* extract had the strongest antiviral activity against PRRSV replication. These activities can be presumably attributed to the total phenolic contents and antioxidant activities of these plant extracts. Although several previous studies have shown the antiviral activity of plant extracts against PRRSV, there are no reports on the antiviral activities of *T. triandra* and *C. sappan* extracts against PRRSV. To the best of our knowledge, this study is the first to report the inhibitory activity of *T. triandra* and *C. sappan* extracts against PRRSV activity in vitro. Further studies are required to elucidate the mechanisms of action of these plant extracts on PRRSV.

## Methods

### Chemicals

All chemicals used in this study were of analytical grade or higher. Ethanol and methanol were obtained from Merck (Darmstadt, Germany). ABTS, 6-hydroxy-2,5,7,8-tetramethylchroman-2-carboxylic acid (Trolox), DPPH, Folin–Ciocalteu phenol reagent, 3-(4,5-dimethylthiazol-2-yl)-2,5-diphenyl tetrazolium bromide (MTT), sodium carbonate, and 2,4,6-tri-pyridyl-s-triazine were purchased from Sigma Chemical Co. (St. Louis, MO, USA). Ferric chloride hexahydrate and potassium persulfate were procured from LOBA CHEMIE PVT (Mumbai, India). Gallic acid was procured from Fluka Chemical Co. (Buchs, Switzerland). Dulbecco’s modified Eagle’s medium (DMEM) was procured from Gibco (Massachusetts, USA).

### Plant extracts, cells, and viruses

Ethanolic *C. sappan*, *G. mangostana*, *H. cordata*, *P. frutescens*, *C. nutans*, *P. emblica*, and *T. triandra* extracts were purchased from Specialty Natural Product Co. Ltd. (Thailand).

MARC-145 tissue culture cells were grown in DMEM containing 10% fetal bovine serum (Gibco) and 1% penicillin/streptomycin and incubated at 37 °C in a 5% CO_2_ atmosphere. To produce inoculated cells, PRRSV (VR2332 North American genotype) was propagated in MARC-145 cells, and virus titer was quantified using IPMA.

### Cytotoxicity assay

The cytotoxicity of the seven Thai medicinal plant extracts was determined using the MTT assay. Briefly, MARC-145 cells were plated at a density of 5000 cells/well in 96-well plates and incubated in a 5% CO_2_ atmosphere at 37 °C for 24 h. When cells had at least 90% confluence, the medium was removed and replaced with medium containing two-fold serial dilutions of the plant extracts. In addition, medium without plant extract was used as a positive control. Incubation was then continued in a 5% CO_2_ atmosphere at 37 °C for 72 h. After this, the medium was removed, 20 μl of freshly prepared MTT solution (5 mg/ml) was added to each well, and the plates were incubated at 37 °C for 4 h. Then, the medium was replaced with 150 μl DMSO to dissolve the crystals, and the plates were incubated at 37 °C for 5 min to dissolve any air bubbles before measuring the MTT signal at an absorbance of 550 nm. Results are reported as CC_50_.

### Inhibition of virus infection assay

The inhibition of virus infection assay was performed as previously described [12]. Briefly, the plant extracts at the concentration that was determined in the cytotoxicity test outlined above and at two lower concentrations in two-fold dilution were mixed with PRRSV at 10^8^ TCID_50_/ml at a ratio of 1:1 and incubated at 37 °C for 1 h. DMSO (1%) containing medium mixed with PRRSV served as the control. Thereafter, the mixture of PRRSV and plant extracts as well as controls were inoculated in MARC-145 cells at a density of 5000 cells/well in a 96-well plate and incubated at 37 °C for 1 h. Subsequently, the medium was removed and replaced with a fresh medium containing 10% FBS. The plates with MARC-145 cells were cultured under standard conditions for 24 h hpi, and supernatants were collected to quantify virus titer.

### Inhibition of viral replication assay

The inhibition of viral replication assay was performed as previously described [12]. Briefly, MARC-145 cells were plated at a density of 5000 cells/well in 96-well plates and infected with PRRSV at a multiplicity of infection of 1 at 37 °C for 1 h. Then, PRRSV was removed from each well and replaced with the diluted plant extracts at the concentration that was determined in the cytotoxicity test and at two lower concentrations ins two-fold dilution. Further, 1% DMSO was mixed to medium as the control. The plates were cultured under standard conditions; supernatants were collected at 24, 48, and 72 hpi; and virus titer was quantified.

### Virus titer

Virus titer was further assessed by IPMA as previously described [30]. Briefly, cells were fixed with 100 μl of 4% cold formalin for 15 min at room temperature (RT), washed once with 100 μl of phosphate-buffered saline (PBS) and twice with 100 μl of 0.5% PBS Tween-20 (PBST), and blocked with 100 μl of 1% BSA in 0.5% PBST for 30 min at RT. After blocking, the cells were stained with 70 μl of anti-PRRSV NC protein monoclonal antibody (Median Diagnostics, Gangwon-do, Korea) diluted at a ratio of 1:400 at RT for 60 min, washed, and incubated with peroxidase-conjugated AffiniPure Goat Anti-Mouse IgG (H + L) (Jackson ImmunoResearch, Pennsylvania, USA) diluted at a ratio of 1:1200 for 60 min at RT. After washing thrice with PBS, the cells were counter stained with 1,5-diaminopentane substrate and examined under a microscope. Virus titer is expressed as TCID_50_ and was determined using the Reed–Muench method.

### Phytochemical analysis

The total phenolic contents of the plant extracts were determined using the Folin–Ciocalteu method [31], and their free radical-scavenging activities were determined using the DPPH-scavenging and ABTS-scavenging assays, as previously reported [32, 33]. Antioxidant activities were determined using the FRAP assay, according to the Benzie and Strain method [34].

### Statistical analysis

Differences in antiviral activities among the different concentrations of each plant extract were tested using one-way analysis of variance with Tukey’s post hoc test for a comparison of means. CC_50_ was calculated using regression analysis of dose–response curves for the MTT assay. All statistical analyses were performed using the SPSS 23.0 software (SPSS Inc., Chicago, IL, USA) with a significance level of *P*-value of ≤0.05.

## Data Availability

The datasets supporting the results of this article are available in the figshere (https://figshare.com/s/97bfdb8d693a8c95ffaf).

## Abbreviations

PRRS: Porcine reproductive and respiratory syndrome
PRRSV: Porcine reproductive and respiratory syndrome virus
TCID_50_: Median tissue culture infective dose
RNA: Ribonucleic acid
hpi: Hour post-infection
CC_50_: 50% cytotoxic concentration
IPMA: Immunoperoxidase monolayer assay
GAE: Gallic acid equivalent
DPPH: 2, 2-diphenyl-1-picrylhydrazyl
ABTS: 2, 2-azino-bis(3-ethylbenzothiazo-line-6-sulfonic acid) diammonium salt
FRAP: Ferric-reducing antioxidant power
IC_50_: Half-maximal inhibitory concentration
DMEM: Dulbecco’s Modified Eagle Medium
MTT: 3-(4,5-dimethylthiazol-2-yl)-2,5-diphenyl tetrazolium bromide
CO_2_: Carbon dioxide
DMSO: Dimethyl sulfoxide
FBS: Fetal bovine serum
PBS: Phosphate-buffered saline
RT: Room temperature
